# In our own image? Emotional and neural processing differences when observing human–human *vs* human–robot interactions

**DOI:** 10.1093/scan/nsv043

**Published:** 2015-04-23

**Authors:** Yin Wang, Susanne Quadflieg

**Affiliations:** Division of Psychology, New York University|Abu Dhabi, Abu Dhabi, UAE

**Keywords:** impression formation, mind attributions, person construal, person dyads, social robotics

## Abstract

Notwithstanding the significant role that human–robot interactions (HRI) will play in the near future, limited research has explored the neural correlates of feeling eerie in response to social robots. To address this empirical lacuna, the current investigation examined brain activity using functional magnetic resonance imaging while a group of participants (*n* = 26) viewed a series of human–human interactions (HHI) and HRI. Although brain sites constituting the mentalizing network were found to respond to both types of interactions, systematic neural variation across sites signaled diverging social-cognitive strategies during HHI and HRI processing. Specifically, HHI elicited increased activity in the left temporal–parietal junction indicative of situation-specific mental state attributions, whereas HRI recruited the precuneus and the ventromedial prefrontal cortex (VMPFC) suggestive of script-based social reasoning. Activity in the VMPFC also tracked feelings of eeriness towards HRI in a parametric manner, revealing a potential neural correlate for a phenomenon known as the uncanny valley. By demonstrating how understanding social interactions depends on the kind of agents involved, this study highlights pivotal sub-routes of impression formation and identifies prominent challenges in the use of humanoid robots.

## INTRODUCTION

Whether we see two lovers sharing an intimate embrace or a group of colleagues discussing a business proposal, decades of social-psychological research suggest that brief glances at interacting others can inform far-reaching conclusions about them. Beyond deducing their interpersonal intentions (e.g. for affiliation or dominance), people’s capacity for agency, empathy and moral reasoning may be inferred based on how they treat each other (Costanzo and Archer, 1989; Fiske, 1992; Gray and Wegner, 2009; Proverbio *et al.*, 2011; Canessa *et al.*, 2012; Gray *et al.*, 2012; Mason *et al.*, 2014). According to recent neuroimaging studies, this feat of drawing complex social inferences from merely looking at person interactions relies on the recruitment of at least two well-defined brain networks—the so-called person perception network (PPN) and the mentalizing network (MTN; Iacoboni *et al.*, 2004; Walter *et al.*, 2004; Pierno *et al.*, 2008; Hooker *et al.*, 2010; Sinke *et al.*, 2010; Centelles *et al.*, 2011; Kujala *et al.*, 2012; Wagner *et al.*, 2011; Spunt and Adolphs, 2014; Quadflieg *et al.*, 2015).

The PPN, usually thought of as comprising the occipital face area (OFA), extrastriate body area (EBA), fusiform face area (FFA), fusiform body area (FBA) and the posterior superior temporal sulcus (pSTS), is known to extract information about people’s facial and bodily appearance (Weiner and Grill-Spector, 2010; Haxby and Gobbini, 2011). The MTN, in contrast, has been argued to implement speculations about people’s beliefs, desires, feelings, motives, or intentions that may explain their visible behavior (Gobbini *et al.*, 2007; Abraham *et al.*, 2008; Spunt and Lieberman, 2012), recruiting the ventral and dorsal medial prefrontal cortex (VMPFC, DMPFC), anterior temporal lobe (aTL), temporal–parietal junction (TPJ) and the precuneus (PrC). Although both networks have attracted extensive scientific scrutiny, the exact functional role of their constituting brain sites remains a matter of debate (Aichhorn *et al.*, 2009; Atkinson and Adolphs, 2011; Hartwright *et al.*, 2014; Satpute *et al.*, 2014).

To further delineate the sites’ contributions in the impression formation process, researchers have begun to probe their responses towards humanoid robots. At the heart of this unorthodox approach lies the idea that encountering robots poses a fascinating social-cognitive dilemma (MacDorman and Ishiguro, 2006; Chaminade and Cheng, 2009). Although their facial and bodily appearance may closely resemble the human form (e.g. Minato *et al.*, 2006; Shaw-Garlock, 2009; Saygin *et al.*, 2012; Hall *et al.*, 2014), robots are widely considered incapable of inner experience and independent thought (Robbins and Jack, 2006; Gray *et al.*, 2007; Bartneck, 2013; Rosenthal-von der Pütten *et al.*, 2014). In consequence, observing them can trigger a human-like response in the PPN (Chaminade *et al.*, 2010; Cheetham *et al.*, 2011; Dubal *et al.*, 2011; Gobbini *et al.*, 2011), but usually results in subdued activity in the MTN (Krach *et al.*, 2008; Carter *et al.*, 2011; Gobbini *et al.*, 2011; Chaminade *et al.*, 2012; Takahashi *et al.*, 2014).

What remains to be investigated is whether commonalities and differences in the neural processing of humans and robots are modulated by the kind of behavior these targets engage in (Turing, 1950; Mori, 1970; Opfer, 2002; Ramsey and Hamilton, 2010). According to recent reports, robots that act too human-like (e.g. by showing emotions or trying to befriend someone) elicit particularly strong discomfort in human perceivers (Dautenhahn *et al.*, 2005; Normile, 2014). This discomfort seems to result from spontaneous mind attributions (Gray and Wegner, 2012). In other words, forming impressions of seemingly self-propelled, goal-directed and sentient robots may activate the MTN in a manner that translates into feelings of eeriness towards them. To examine this interesting possibility, this study investigated perceivers’ emotional, cognitive and neural responses during the observation of a series of human–human interactions (HHI) and human–robot interactions (HRI).

## MATERIALS AND METHODS

### Participants

Twenty-six White native English speakers, aged between 18 and 35 years (mean: 21.7 years, 14 females) participated in the study. All were right-handed as assessed by the Edinburgh inventory (Oldfield, 1971) and reported normal or corrected-to-normal vision. None had a history of neurological or neuropsychiatric disorders or was currently taking psychoactive medication. Written informed consent was obtained from all individuals. The study protocol was jointly approved by the Institutional Review Board of New York University Abu Dhabi and New York University New York.

### Stimuli

Participants completed three tasks while undergoing functional magnetic resonance imaging (fMRI)—an interaction categorization task, a mentalizing localizer and a person perception localizer. During the interaction categorization task, participants viewed a series of color images displaying dyadic social interactions (Quadflieg *et al.*, 2015). The interactions varied along their instrumentality and socio-emotional content (Proverbio *et al.*, 2011). Thus, some were of primarily instrumental value (e.g. giving directions), others fulfilled largely socio-emotional needs (e.g. exchanging a hug), and yet others combined both aspects (e.g. donating money to a beggar) or seemed to lack either (e.g. having a chat). Interactions could further involve one agent acting upon another (e.g. by ‘presenting a gift’ or ‘proposing marriage’) or two agents acting in a reciprocal manner (e.g. by ‘shaking hands’ or ‘sharing a dance’).

**Fig. 1:**
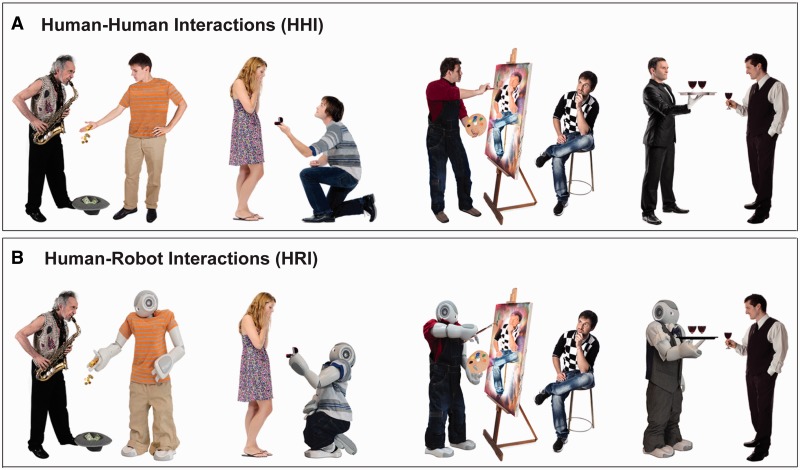
Example images as used in the interaction categorization task. Participants viewed 40 different dyadic interactions throughout the task. Per run, each interaction was portrayed once as a human–human encounter and once as a human–robot encounter.

For each interaction, a human–human version and a human–robot version were created. To do so, 40 HHI unfolding between two White individuals (i.e. between two individuals of the perceivers’ racial ingroup) were downloaded from shutterstock® Photos (www.shutterstock.com), equalized in height, and embedded in a uniform white background of 400 × 400 pixels (visual angle during presentation: 15° × 15°). Subsequently, corresponding HRI were created by replacing one of the two original human interaction partners with a humanoid robot called Nao (http://www.aldebaran-robotics.com). Nao was photographed in postures and clothing that resembled its relevant human counterparts (all of which were male). The resulting photographs were then digitally optimized to approximate the original model’s height, pose and outfit using Adobe Photoshop© (Version 12.0.4; see Figure 1 and Supplementary Material).

For the mentalizing localizer, 20 short stories as previously used in the field (Dodell-Feder *et al.*, 2011) were downloaded from http://saxelab.mit.edu/superloc.php. Half of the stories described false beliefs, whereas the other half described false photographs, signs and maps. Matched on logical complexity, only mental state stories but not physical state stories required a reader to build a representation of someone else’s belief. All stories were presented centrally in White Arial Font (40 pt) against a uniform black background. Finally, during the person perception localizer, 42 human faces (21 female), 42 human bodies (21 female) and 42 cars, as well as phase-scrambled controls for faces and bodies were used (taken from Quadflieg *et al.*, 2011). Stimuli were presented in color on a uniform grey background, standardized to a common size [184 (width) × 210 (height) pixel]. Faces and bodies used in the localizer task were not presented at any other point during the study.

### fMRI task and procedure

Participants were invited to take part in a study about the neural mechanisms of perceiving social interactions. They were informed that they would be shown a series of images displaying social interactions which involved either two humans or a human and a robot. For each interaction, they were asked to indicate whether one agent was seen helping the other via a button press with their right hand (i.e. yes = index finger, no = middle finger). They were also informed that some interactions would seem easier to judge than others and that they should rely on their intuitive response instead of overthinking their decision. A helping judgment was requested for two main reasons: first, it ensured that perceivers held an identical processing goal throughout the task, regardless of interaction type. Second, it encouraged perceivers to process both agents per interaction by probing their relation towards each other.

The interaction categorization task was set up as a rapid event-related fMRI experiment. It comprised two separate runs, each lasting about 9 min. In both runs, participants encountered the same 40 HHI and 40 HRI, but each time in a new pseudo-random order. On each trial, an interaction appeared on a white background. After 2000 ms ( = 1 repetition time (TR)) it was replaced by a black fixation cross. The duration of the fixation cross was a multiple of the TR and lasted between 2000 and 12 000 ms, causing varying inter-stimulus intervals throughout each run (Ollinger *et al.*, 2001). The duration of fixations and order of stimuli was optimized using optseq2 (http://surfer.nmr.mgh.harvard.edu/optseq/). Four different optimized sequences were used in a counterbalanced manner across runs and participants.

Localizer tasks were set up as previously described in the literature (see also Supplementary Material). In short, the mentalizing localizer (cf. Koster-Hale *et al.*, 2013) comprised one run, lasting approximately 9 min. During this time, participants were asked to read the selected mental and physical stories. Story comprehension was probed by a true/false statement following each story. The person perception localizer (cf. Quadflieg *et al.*, 2011) comprised three runs, each lasting about 10 min. During this time, participants viewed blocks of consecutively presented images and performed a 1-back repetition detection task. The order of all experimental runs was also fixed, such that participants completed two runs of the categorization task, one run of the mentalizing localizer and three runs of the person perception localizer. Stimuli were back projected onto a screen visible via a mirror mounted on the MRI head coil. Stimulus presentation and recording of participants’ responses were accomplished using Presentation® software (Neurobehavioral Systems Inc.) and Cogent 2000 (University College London Functional Imaging Laboratory).

After scanning a Qualtrics online survey was administered to all participants, using a MacBook Pro laptop equipped with a 15 inch screen. In this survey, the same interactions as in the scanner were shown, but in a new randomized order. This time, participants were required to rate how eerie as well as how believable each interaction seemed (1 = not at all to 7 = very much). In addition, an asterisk marked the agent of each dyad that appeared once as a robot and once as a human throughout the study. For these designated targets, participants additionally rated how intelligent and how capable of emotions they looked (1 = not at all to 7 = very much), capturing their inclination to attribute a human-like mind to these targets (Gray *et al.*, 2007).

### Image acquisition

Image acquisition was undertaken on a 3 Tesla head scanner (Siemens Allegra, Erlangen, Germany) with an eight channels array head coil. Functional images were collected using a T2*-weighted gradient echo planar imaging (EPI) sequence (TR = 2000 ms, echo time (TE) 30 ms, flip angle = 82°, 3 × 3 in-plane resolution; field of view 240 mm; acquisition matrix 64 × 80). For each volume, 35 axial slices parallel to the bi-commissural line (anterior commissure − posterior commissure) with 3 mm slice thickness and 0 mm skip between slices were acquired. For each participant, 245 volumes for each run of the interaction categorization task were collected, 284 volumes for each run of the person perception localizer, and 267 volumes for the mentalizing localizer. To account for T1 saturation effects, the first four volumes of each run were discarded.

### Data analysis

Behavioral data were analyzed using SPSS for Windows. For statistical analyses of the fMRI data SPM8 (Wellcome Department of Imaging Neuroscience, London, UK) was used. Standard fMRI data preprocessing began by slice-time correcting the functional data to the middle slice of each whole-brain volume. Subsequently, the functional data were realigned and unwarped using a least square approach and a six parameter (rigid body) spatial transformation. Following realignment, the mean EPI image was normalized to the standard EPI template. In addition, all functional data were spatially smoothed (6 mm full-width-half-maximum Gaussian kernel). After these standard fMRI data preprocessing steps, three types of statistical analyses were conducted.

First, an exploratory univariate whole-brain analysis examined the effects of interaction type in the categorization task. Thus, a two-run event-related design was modeled using a canonical hemodynamic response function (HRF) with two regressors of interest (HHI *vs* HRI) and a 100 s high pass temporal filter. Contrast effect maps (HHI > HRI) were computed for each participant and then entered into a second-level repeated measures analysis of variance (ANOVA), treating participants as a random effect. To minimize false-positive results, effects were considered statistically significant using a voxelwise threshold of *P* < 0.005, a cluster-based threshold of *P* < 0.05 (false discovery rate (FDR) corrected).

Second, a region of interest (ROI) approach was adopted to investigate the neural processing of HHI and HRI. For the mentalizing localizer, a one-run block design was modeled using a canonical HRF to create two regressors of interest (mental states *vs* physical states) and a 128 s high-pass temporal filter. For the person perception localizer, a three-run block design was modeled using a canonical HRF to create regressors of interest (faces, scrambled faces, bodies, scrambled bodies and cars) and a 160 s high-pass filter. High-pass filters were chosen based on the maximum time of repetition between trials of the same type within each task (cf. Skudlarski *et al.*, 1999; Goebel *et al.*, 2006). In a next step, statistical parametric maps were computed for each participant and each regressor of interest against baseline. Subsequently, ROIs constituting the PPN and MTN were identified for each participant. To isolate brain areas responding preferentially to human faces (i.e. OFA, FFA, pSTS), the contrast faces > cars was masked with the contrast faces > scrambled faces. To isolate areas responding to human bodies (i.e. EBA, FBA), the contrast bodies > cars was masked with the contrast bodies > scrambled bodies. To isolate mentalizing ROIs (i.e. VMPFC, DMPFC, aTL, TPJ and PrC), mental state stories > physical state stories was computed. All ROIs were specified as a set of contiguous voxels significantly activated (*P* < 0.05, uncorrected) within a 9 mm cube surrounding a relevant region-specific peak voxel to ensure that ROIs could be segregated from nearby activations (Peelen *et al.*, 2006). Subsequently, parameter estimates for HRI and HHI in each ROI were extracted based on the statistical parametric maps created for the whole-brain analysis, using ‘MarsBaR’ (Brett *et al.*, 2002).

Third, to identify brain regions associated with perceivers’ post-scanning interaction ratings, a set of parametric analyses were run. Thus, for each participant, their unique (mean-centered) post-scanning ratings were assigned as trial-specific modulation parameters. Given participants had provided four different ratings, four separate models were built per interaction type, each comprising one parametric modulator. Statistical parametric maps were computed for each participant and entered into a second-level repeated measures ANOVA, treating participants as a random effect. Criteria to minimize false-positive results for parametric analyses were the same as for the whole-brain analysis.

## RESULTS

### Interaction categorization performance

Analyzing participants’ mean response times on the interaction categorization task revealed that HRI and HHI were categorized equally quickly [*M*_HRI_ = 1127, s.d._HRI_ = 109; *M*_HHI_ = 1113, s.d._HHI_ = 97; *t*(25) = 1.38, *P* = 0.18, *d* = 0.27]. Although HRI and HHI elicited similar helping decisions at large (percentage of agreement across corresponding interactions per run: *M* = 90.77%; s.d. = 5.65%), HRI were seen as involving slightly more helping than HHI (*M*_HRI_ = 54%, s.d._HRI_ = 8; *M*_HHI_ = 51%, s.d._HHI_ = 8; *t*(25) = 3.83, *P = *0.001, *d* = 0.75].

### Post-scanning ratings

Submitting participants’ average post-scanning rating scores to a series of paired *t*-tests revealed significant differences on all dimensions probed. HRI were generally seen as ‘eerier’ than HHI [*M*_HRI_ = 3.16, s.d._HHI_ = 1.38; *M*_HHI_ = 1.40, s.d._HHI_ = 0.46; *t*(25) = 5.94, *P* < 0.001, *d* = 1.16]. In addition, participants found HRI less ‘believable’ than HHI [*M*_HRI_ = 3.75, s.d._HRI_ = 1.40; *M*_HHI_ = 5.89, s.d._HHI_ = 0.86; *t*(25) = 6.79, *P* < 0.001, *d* = 1.33]. Moreover, they perceived robotic targets as less ‘capable of emotions’ [*M*_HRI_ = 3.68, s.d._HHI_ = 1.38; *M*_HHI_ = 6.20, s.d._HHI_ = 0.65; *t*(25) = 8.54, *P* < 0.001, *d* = 1.67] and less ‘intelligent’ [*M*_HRI_ = 4.29, s.d._HHI_ = 1.38; *M*_HHI_ = 5.45, s.d._HHI_ = 0.82; *t*(25) = 3.91, *P* = 0.001, *d* = 0.77] than their human counterparts.

We also examined whether perceivers’ feelings of eeriness during interaction perception were associated with their believability and mind prevalence assessments. Thus, for each participant, we calculated the relevant correlation coefficients across all scenarios of the same interaction type. The average of these correlation coefficients across participants was then tested against zero. For HHI, it was found that eeriness increased, the less believable they seemed [*M*_r_ = −0.24, s.d._r_ = 0.29; *t*(25) = 4.27, *P* < 0.001, *d* = 0.84]. In addition, increases in eeriness were associated with decreases in a person’s alleged emotionally capacity [*M*_r_ = −0.19, s.d._r_ = 0.26; *t*(25) = 3.68, *P* = 0.001, *d* = 0.72] or intelligence [*M*_r_ = −0.16, s.d._r_ = 0.18; *t*(25) = 4.66, *P* < 0.001, *d* = 0.91]. Similarly, for HRI, increases in eeriness were accompanied by decreases in believability [*M*_r_ = −0.40, s.d._r_ = 0.24; *t*(25) = 8.44, *P* < 0.001, *d* = 1.66]. Eeriness failed to correlate, however, with a robot’s alleged emotional capacity [*M*_r_ = 0.12, s.d._r_ = 0.33; *t*(25) = 1.82, *P* = 0.08, *d* = 0.36] or intelligence [*M*_r_ = 0.01, s.d._r_ = 0.26; *t*(25) = 0.17, *P* = 0.87, *d* = 0.03].

To directly compare the diverging correlation patterns for HHI and HRI, we also submitted participants’ correlation coefficients to a series of paired *t*-tests. Doing so revealed that the link between believability and eeriness was significantly weaker in HHI than HRI [*t*(25) = 2.83, *P* = 0.009, *d* = 0.56]. In contrast, the link between emotional capacity and eeriness was significantly stronger in HHI than HRI [*t*(25) = 3.96, *P* = 0.001, *d* = 0.78], as was the link between intelligence and eeriness [*t*(25) = 2.76, *P* = 0.011, *d* = 0.54].

### Whole-brain fMRI analyses

**Fig. 2:**
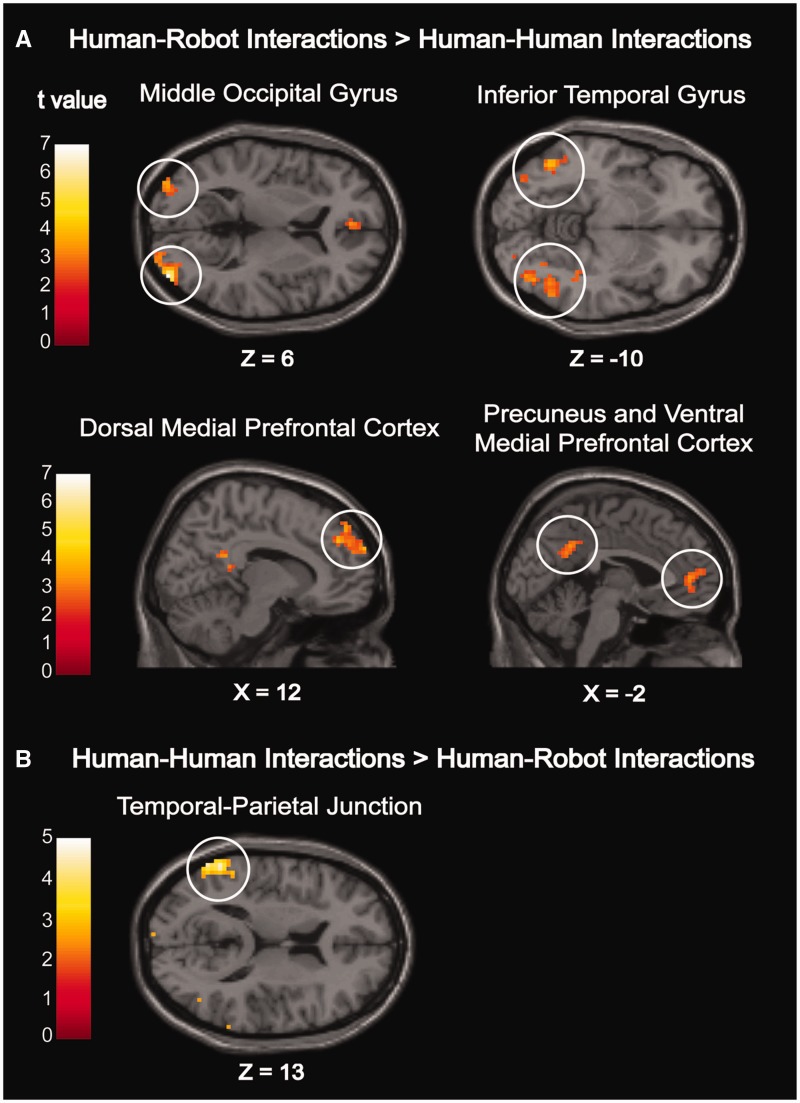
Cortical activations during the interaction categorization task as determined by a whole-brain analysis at a voxelwise threshold of *P* < 0.005 and a cluster-size threshold of *P* < 0.05 (FDR corrected).

Exploratory univariate whole-brain analyses were undertaken to examine the effects of interaction type in the interaction categorization task (Table 1, Figure 2). The contrast HHI > HRI revealed an enhanced response in the left TPJ. The reverse contrast HRI > HHI yielded enhanced activity in the bilateral middle occipital gyrus, bilateral inferior temporal cortex (extending into the fusiform and the inferior occipital gyrus), medial PrC, as well as in the DMPFC and the VMPFC.

**Table 1:** Peak voxel in MNI coordinates and number of voxels for brain regions as identified from the interaction categorization task by whole-brain analyses at a voxelwise threshold of *P* < 0.005 and a cluster-size threshold of *P* < 0.05 (FDR corrected)

Region	Hemisphere	Voxels	*T*	*P*-value	*x*	*y*	*z*
HHI > HRI							
TPJ	L	126	5.21	0.005	−60	−52	13
HRI > HHI							
Middle occipital gyrus	R	162	8.33	0.001	39	−91	7
	L	87	4.68	0.011	−36	−94	4
Inferior temporal gyrus	R	99	6.44	0.009	54	−58	−11
	L	74	4.71	0.017	−48	−61	−8
PrC	Midline	144	4.84	0.002	9	−49	22
DMPFC	Midline	86	4.67	0.011	12	44	34
VMPFC	Midline	55	3.91	0.045	−3	47	7

### Localizer-based fMRI analyses

**Fig. 3:**
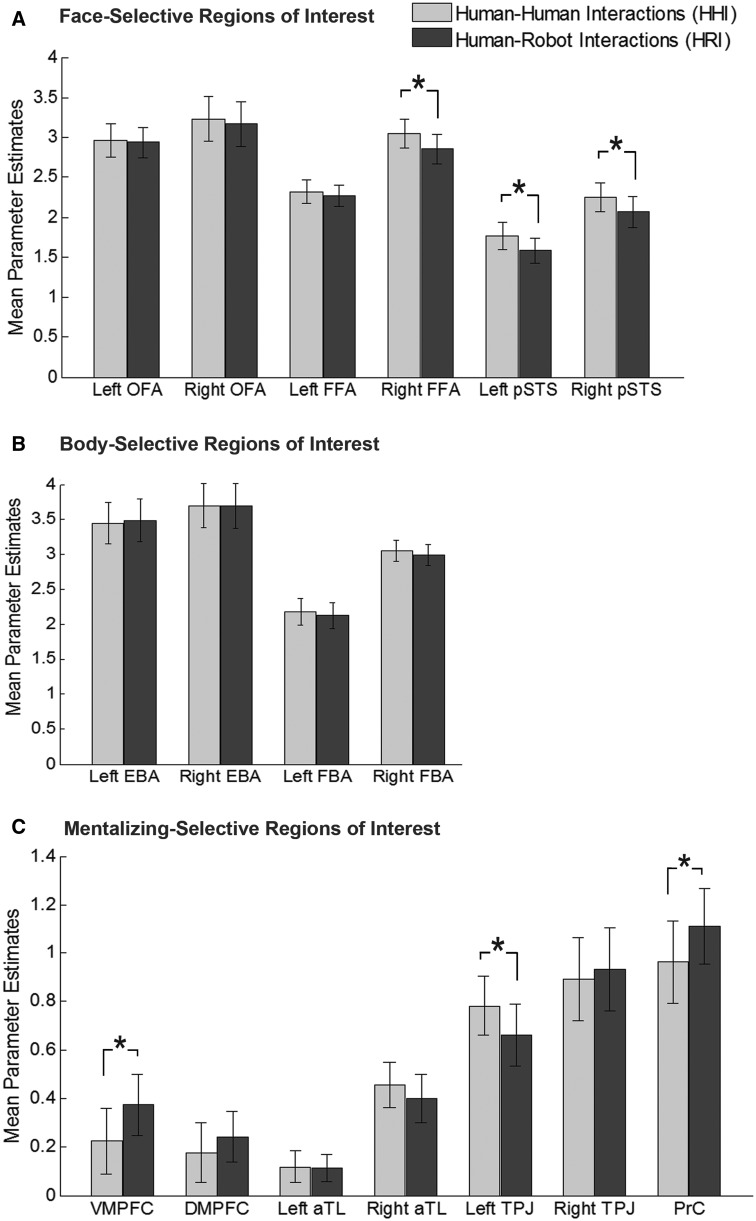
Neural responses during the interaction categorization task in regions of interest as determined by a person perception localizer and a mentalizing localizer.

Table 2 lists the average peak Montreal Neurological Institute (MNI) coordinates of all ROIs across participants, including the number of individuals for which each ROI was identified. The regions are in agreement with previous work using the same localizers (cf. Dodell-Feder *et al.*, 2011; Quadflieg *et al.*, 2011). Mean parameter estimates in all ROIs were extracted from the interaction categorization task for each participant and submitted to a series of paired *t*-tests (Figure 3). For mentalizing ROIs, an effect of interaction type was found in three ROIs. Stronger activation for HHI than HRI emerged in the left TPJ [*t*(24) = 2.23, *P* = 0.035]. Stronger activations for HRI than HHI emerged in the VMPFC [*t*(22) = 2.39, *P* = 0.026] and the PrC [*t*(24) = 2.33, *P* = 0.028]. No significant effects were observed in other ROIs of the MTN (*t’*s < 1.22, *P’*s > 0.23). For face-selective ROIs, stronger activations for HHI *vs* HRI were found in the right FFA [*t*(24) = 3.48, *P* = 0.002] and bilaterally in the pSTS [right: *t*(25) = 3.18, *P* = 0.004; left: *t*(22) = 3.10, *P* = 0.005]. In the remaining face- and body-selective ROIs, no significant activity differences emerged (all *t’*s < 1.12, *P’*s > 0.27).

**Table 2:** Mean MNI coordinates of person perception and mentalizing ROIs as determined based on the corresponding localizer tasks

Region	Hemisphere	*N*	*x*	*y*	*z*
Face-selective regions of interest					
OFA	R	24	42	−79	−13
	L	19	−40	−80	−14
FFA	R	25	43	−50	−24
	L	26	−40	−49	−23
pSTS	R	26	53	−54	11
	L	23	−51	−59	12
Body-selective regions of interest					
EBA	R	25	48	−77	−1
	L	25	−49	−78	4
FBA	R	25	43	−49	−23
	L	24	−42	−49	−21
Mentalizing regions of interest					
aTL	R	25	52	4	−34
	L	25	−49	3	−35
TPJ	R	25	53	−55	21
	L	25	−50	−59	21
DMPFC	Midline	23	1	54	33
VMPFC	Midline	23	1	54	−8
PrC	Midline	25	1	−57	37

### Parametric fMRI analyses

**Fig. 4:**
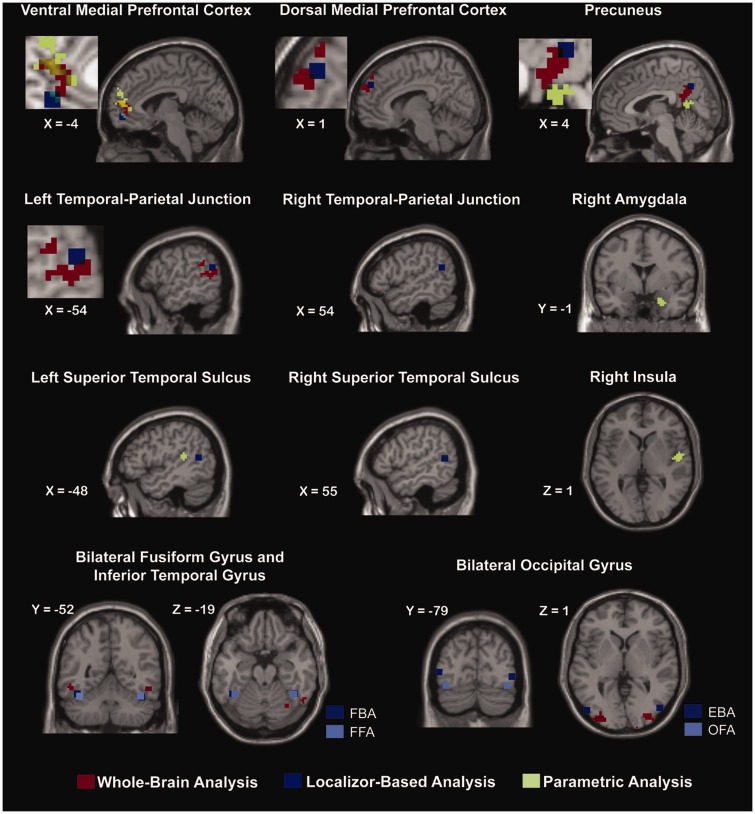
Neural activity as revealed by the three different analyses. Localizer-based regions of activity are displayed as 9 mm cubes plotted around the average peak MNI coordinate across all participants. Note that partial overlap was observed across at least two of the three analyses in the VMPFC, the DMPFC, the PrC, and the left TPJ.

Parametric analyses were limited to perceivers who showed actual variation in their post-scanning ratings for both HHI and HRI (i.e. believability: *n* = 22; eeriness: *n* = 23; emotional capacity: *n* = 24; intelligence: *n* = 25). For HHI, all parametric analyses failed to return significant results. For HRI, in contrast, several parametric modulations were found. Specifically, increases in eeriness were associated with enhanced VMPFC activity during HRI observation [peak voxel *x* = 9, *y* = 53, *z* = 16; *t* = 5.31, *P* < 0.001, *P*(FDR) = 0.006, 142 voxels]. In addition, increases in believability were linked to enhanced PrC activity [peak voxel *x* = − 15, *y* = −58, *z* = 22; *t* = 6.28, *P* < 0.001, *P*(FDR) < 0.001, 189 voxels]. Finally, the higher a robot’s perceived emotional capacity, the stronger perceivers’ neural responses in the right amygdala [peak voxel *x* = 24, *y* = −1, *z* = −26; *t* = 5.64, *P* < 0.001; *P*(FDR) = 0.008, 59 voxels], right insula [peak voxel *x* = 54, *y* = −4, *z* = 1; *t* = 4.72, *P* < 0.001; *P*(FDR) = 0.008, 52 voxels] and the left STS [peak voxel *x* = −51, *y* = −37, *z* = 13; *t* = 5.26, *P* < 0.001, *P*(FDR) = 0.020, 39 voxels]. Only for intelligence ratings, parametric modulation of brain activity during HRI viewing failed to emerge. To compare these results with those from previous analyses, we plotted them in a common graph (Figure 4). Repeating the parametric analyses with all participants included revealed highly similar, albeit slightly less significant, effects (see Supplementary Material).

## DISCUSSION

Accumulating evidence suggests that observing robots compared with humans results in diminished MTN engagement (Krach *et al.*, 2008; Carter *et al.*, 2011; Gobbini *et al.*, 2011; Takahashi *et al.*, 2014). This neural difference has been argued to reflect perceivers’ divergent outlooks on robots and people, with the former being generally expected to lack agency and inner experience (Gray *et al.*, 2007). Initial data suggest, however, that witnessing ostensibly self-propelled and goal-directed behavior can tempt perceivers to attribute a human-like mind even to non-human entities, including robots (Turing, 1950; Opfer, 2002; Ramsey and Hamilton, 2010). Yet, such attributions may come at the prize of feeling eerie towards them (Gray and Wegner, 2012). To examine this interesting possibility, we asked a group of participants to observe and evaluate a series of social interactions unfolding either between two humans (HHI) or between a human and a robot (HRI).

As expected, HRI elicited stronger feelings of eeriness than HHI at large. But in conflict with our predictions, variation in eeriness across the different HRI failed to be associated with perceivers’ spontaneous mind attributions. Only for HHI, increases in eeriness were accompanied by systematic decreases in perceived mind capacities. For HRI, in contrast, the two processes seemed largely unrelated (see also Broadbent *et al.*, 2013). In other words, the portrayal of different kinds of social behaviors successfully induced varying levels of eeriness towards both HHI and HRI, but this variation was unaccounted for by mind attributions in the latter case. Future work should therefore explore the role of alternative explanatory variables as previously discussed, such as interaction-specific concerns about a robot’s potential to harm others (Tinwell, 2014) or to get harmed by them (Misselhorn, 2009).

Despite the lack of association between eeriness and mind attributions for HRI, this study found that perceivers relied less strongly on mind attributions overall when interpreting the actions of robots compared with humans. In addition, interactions involving robots were more frequently understood to portray helping than interactions exclusive to humans. In concert, the denial of a subjective mind and the preferential construal of actions based on their utility signals stronger objectification of robots than humans (Nussbaum, 1995). Moreover, social interactions between a robot and a human were considered less believable than similar interactions between two people, providing further evidence that participants adopted diverging social-cognitive strategies during HRI and HHI processing.

This conclusion was also supported by the obtained neuroimaging findings. A whole-brain analysis revealed that HHI elicited enhanced activity in the left TPJ, whereas HRI yielded increased activity in the DMPFC and VMPFC, the medial PrC, as well as in the bilateral middle occipital gyrus and inferior temporal gyrus. A subsequent localizer-based analysis showed that differences in the left TPJ, PrC and VMPFC overlapped with brain regions constituting the MTN. Thus, instead of HHI processing being characterized by general MTN enhancement, both HHI and HRI were found to recruit the MTN, but in a differential manner. In doing so, the current findings strengthen the claim that different sites of the MTN have distinct functional roles in the impression formation process (Aichhorn *et al.*, 2009; Hartwright *et al.*, 2014; Satpute *et al.*, 2014).

According to various neuroimaging meta-analyses, bilateral TPJ activity plays a prominent role during mental state deductions (Van Overwalle, 2009; Bzdok *et al.*, 2012; Schurz *et al.*, 2014). Activity in the left TPJ has specifically been associated with inferring invisible beliefs and intentions from other people’s actions (Schurz *et al.*, 2014). In line with this observation, left—but not right—TPJ activity has recently been found to underlie judgments of why (instead of how) two agents are interacting (Spunt and Adolphs, 2014). Moreover, damage to the left TPJ has been shown to cause highly selective deficits in false belief reasoning (Apperly *et al.*, 2004; Samson *et al.*, 2004). Finally, an enhanced proneness to rely on mental states even when explaining the behavior of non-human entities (such as animals or objects) has been linked to enhanced grey matter volume in the left TPJ (Cullen *et al.*, 2014). In concert, these data suggest that the region is critically involved in representing invisible mental states. This study adds to this line of research, revealing that left TPJ activity is systematically reduced when perceivers process interactions that involve at least one mind-deficient interaction partner (i.e. during HRI processing).

The medial PrC and VMPFC, in contrast, responded more strongly during HRI than HHI processing. Both regions are known to foster impression formation by providing access to generalizable social knowledge (Mitchell *et al.*, 2005; Szczepanski and Knight, 2014). Activity in the PrC, for instance, has been linked to the retrieval of stereotypic beliefs about people (Simmons *et al.*, 2010; Contreras *et al.*, 2012; Fairhall *et al.*, 2014). In addition, activity in the VMPFC has been found to support the retrieval of script-based knowledge (van Kesteren *et al.*, 2012; Ghosh *et al.*, 2014), including social script knowledge, as probed by many of our interactions (e.g. giving directions, proposing marriage). In combination, these data suggest that HRI processing elicited more abstract social reasoning than HHI processing. This conclusion converges with recent behavioral reports according to which humans easily detect and even respond to robots’ social behavior by adopting a rule-based communicative point of view (i.e. Which type of response does such behavior normally require?) instead of an experiential approach (i.e. Which emotions/thoughts may have caused this behavior?; Dautenhahn, 2007; Knobe and Prinz, 2008; Krämer *et al.*, 2012; Shariff and Tracy, 2011; Beck *et al.*, 2012).

The results of our parametric analyses further revealed that activity increases as observed in the VMPFC and PrC during HRI viewing were associated with perceivers’ post-scanning ratings of these interactions. Activity in the VMPFC increased more strongly towards HRI, the stronger perceivers’ feelings of eeriness towards them. Activity increase in the PrC, in contrast, was accompanied by a systematic increase in HRI’s believability (albeit this parametric activity pattern was observed in a region located more inferior than the ones identified by the main contrast and the mentalizing localizer; Figure 4). Activity in neither of the two regions, however, tracked perceivers’ spontaneous mind attributions. Instead, attributions of emotional capability elicited parametric modulations of brain activity in areas well known to implement the encoding of emotional states, such as the amygdala, the insula, and the pSTS (see Adolphs, 2002; Haxby and Gobbini, 2011).

Although both our behavioral and neuroimaging findings indicate that perceivers relied on different social-cognitive strategies while viewing HHI and HRI, it must be considered that factors other than the presence of a robot per se might have encouraged these differences. It seems likely, for instance, that perceivers are much more familiar with HHI than HRI. Trying to make sense of unfamiliar social scenes, in turn, might encourage more abstract social reasoning, regardless of the type of agents involved. Future research should therefore aim to include uncommon HHI (e.g. interracial interactions, Pryor *et al.*, 2012) to disentangle the effects of mere familiarity from those specific to non-human agents. Similarly, the observed perceptual processing differences in this study must be considered in further detail. Although HHI elicited enhanced activity in brain sites involved in the encoding of human faces (i.e. right FFA, bilateral pSTS), HRI triggered increased activity in regions dedicated towards perceptual processing outside the PPN (i.e. the middle occipital gyrus and the inferior temporal gyrus). Therefore, variations in high-level social-cognitive strategies may also have resulted from more basic perceptual differences across the two types of interactions.

The robots’ lack of facial detail, expressivity and human-like gaze, for instance, may have produced a shortage of bottom-up perceptual signals upon which to base elaborate mind attributions (Pierno *et al.*, 2008; Saggar *et al.*, 2014; Tinwell *et al.*, 2014). Processing resources beyond the PPN might then have been recruited to detect and encode alternative perceptual signals in a compensatory and more effortful manner (cf. Chaminade *et al.*, 2010). To elucidate the potential impact of early perceptual processes on social-cognitive operations more fully, future work should explore participants’ eye movements and fixation points during HHI and HRI viewing. Equally deserving of further inquiry is the observed lack of parametric modulation of neural activity during HHI processing. Note that participants reported different degrees of mind attributions, believability and eeriness for HHI upon being prompted to do so. Yet, their spontaneous emotional and cognitive responses towards these images may not have used these dimensions to distinguish between them. Instead, HHI (and also HRI) may have invited mind attributions beyond ascriptions of emotional capability and intelligence (e.g. attributions of desires, intentions, or goals) that remained uncaptured by the current investigation.

In recent years, engineering that involves the conception, manufacture and operation of robots has experienced considerable growth (Normile, 2014). As a result, artificial agents once predominantly utilized for military or manufacturing tasks are envisioned to provide domestic, educational, medical and therapeutic support in the near future (Nourbakhsh, 2013). Despite these developments, limited research has explored the neural correlates of prominent feelings of eeriness in the presence of social robots. To address this empirical lacuna, the current investigation explored people’s responses towards robots and people engaged in various kinds of dyadic interactions. Although brain sites constituting the MTN were sensitive to both HHI and HRI, systematic neural variation within the network indicated diverging social-cognitive strategies across the two types of interactions. In addition, activity in the VMPFC tracked feelings of eeriness towards HRI in a parametric manner, revealing a potential neural correlate of the uncanny valley. In summary, these findings demonstrate that even robots outwardly capable of acting like humans elicit emotional, cognitive and neural responses that are remarkably different from those reserved for actual conspecifics.

## SUPPLEMENTARY DATA

Supplementary data are available at *SCAN* online.

## Conflict of Interest

None declared.

## Supplementary Material

Supplementary Data
