# Supplementary material for: In our own image? Emotional and neural processing differences when observing human–human vs human–robot interactions
Source: Soc Cogn Affect Neurosci. 2015 Apr 23;10(11):1515–24. doi: 10.1093/scan/nsv043 (PMC4631149; doi:10.1093/scan/nsv043)
Supplement: Supplementary Data [file supp_10_11_1515__index.html]

In our own image? Emotional and neural processing differences when observing human–human vs human–robot interactions — In our own image? Emotional and neural processing differences when observing human–human vs human–robot interactions — Supplementary Data 

# In our own image? Emotional and neural processing differences when observing human–human *vs* human–robot interactions

## Supplementary Data

files

**Files in this Data Supplement:**

- Supplementary Data - docx file
