# Supplementary material for: In our own image? Emotional and neural processing differences when observing human–human vs human–robot interactions
Source: Soc Cogn Affect Neurosci. 2015 Apr 23;10(11):1515–24. doi: 10.1093/scan/nsv043 (PMC4631149; doi:10.1093/scan/nsv043)
Supplement: Supplementary Data [file supp_nsv043_scan-14-480-File002.docx]

**Supplementary Material**

Interaction Stimuli

To control for our robot’s inability to move its facial features, all human targets that were replaced by the robot displayed either a neutral or no facial expression (e.g., by turning their head away from the perceiver). In addition, care was taken that HHI and HRI closely resembled each other perceptually (cf. Quadflieg et al., 2015). Specifically, both types of interactions were matched regarding their *absolute inter-agent distance* and *inter-agent center of mass distance* as determined by the image processing toolbox in MATLAB (VersionR2012b, ©The MathWorks, Inc.). Thus, to compute the inter-agent distance of each interaction, we identified for each agent the point that was closest to the other agent. The difference between these two points in the image’s x dimension was then calculated (in pixel) and compared across HHI and HRI using a paired t-test [*M*_HHI_ = 2, *SD*_HHI_ = 38; *M*_HRI_ = 4, *SD*_HRI_ = 39; *t*(39) = 1.54, *p* = .22]. In a next step, we determined for each agent its center of mass before computing the distance (in pixel) between these two sets of coordinates per dyad [*M*_HHI_ = 183, *SD*_HHI_ = 31; *M*_HRI_ = 182, *SD*_HRI_ = 31; *t*(39) = 08, *p* = .78].

Procedure

During the mentalizing localizer (Koster-Hale et al., 2013), participants were asked to read the selected short stories. Story comprehension was probed by a true/false statement following each story. Equal numbers of statements requiring a true and a false response were shown for mental states and physical state stories. Participants categorized each statement by pressing a button with their right hand (i.e., true = index finger, false = middle finger). Each story was presented visually for 10 sec, followed by the true/false statement for 4 sec and finally 12 sec of a blank black screen. Stories were presented in a pseudo-random order with story type order counterbalanced across participants.

During the person perception localizer (Quadflieg et al., 2011), participants viewed blocks of consecutively presented images and performed a 1-back repetition detection task, requiring them to press a button with their index finger for any immediate repetition of the same image. In each run, participants encountered 4 blocks of each of the 5 types of visual stimuli (faces, scrambled faces, bodies, scrambled bodies, cars) resulting in a total of 20 alternating blocks per run. Cars were selected as non-human visual control stimuli in line with previous studies on person perception (e.g., Bentin et al. 1996; Grill-Spector et al., 2004). Each block consisted of 18 stimuli from the same visual category and lasted 18 seconds. One or two out of the 18 stimuli per block were repeated. On each trial, image presentation was varied slightly in location (X = ~5%; Y = ~5%) to prevent participants from basing their decision on the inspection of only small sectors of the images. Between blocks a black fixation cross was shown for 9 s epochs. For each person perception localizer run a fixed, pseudo-randomized block order was presented.

To familiarize participants with all tasks, 8 trials of the interaction categorization task, 4 trials of the mentalizing localizer, and 2 blocks of the person perception localizer (1 face block, 1 scrambled bodies block) were completed on a MacBook Pro laptop equipped with a 15 inch screen prior to scanning. None of the stimuli used during practice were shown in the proper study. In case participants commented on any of the stimuli during the practice, they were encouraged to stay focused on the relevant task and ensured that the experimenter would discuss the study in further detail upon debriefing.

Parametric FMRI Analyses Including All Participants

The parametric analyses returned no significant results for HHI. For HRI, increases in believability were associated with increases in PrC activity [peak voxel x = -15, y = -61, z = 22; *t* = 5.33, *p* < .001, *p*(FDR) < .001, 293 voxels]. In addition, increases in feelings of eeriness were associated with enhanced VMPFC activity [peak voxel x = 3, y = 56, z = 16; *t* = 4.21, *p* < .001; *p*(FDR) = .084, 85 voxels]. Moreover, the higher a robot’s perceived emotional capacity, the stronger the activity in the right amygdala [peak voxel x = 24, y = -1, z = -29; *t* = 4.62, *p* < .001; *p*(FDR) = .091, 59 voxels], right insula [peak voxel x = 54, y = -4, z = 1; *t* = 4.40, *p* < .001; *p*(FDR) = 0.155, 47 voxels] and the left STS [peak voxel x = -51, y = -37, z = 13; *t* = 3.73, *p* < .001, *p*(FDR) = .091, 65 voxels]. For ratings of intelligence, no significant findings emerged.

Bentin S, Allison T, Puce A, Perez E, McCarthy G. 1996. Electrophysiological studies of face perception in humans. J CognNeurosci. 8:551-565.

Grill-Spector K, Knauf N, Kanwisher N. 2004. The fusiform face area subserves face perception, not generic within-category identification. Nat Neurosci. 7:555-562.

Koster-Hale J, Saxe R, Dungan J, Young LL. 2013. Decoding moral judgments from neural representations of intentions. Proc Natl Acad Sci USA. 110:5648-5653.

Quadflieg, S., Gentile, F., & Rossion, B. 2015. The neural basis of perceiving person interactions. Cortex. doi: 10.1016/j.cortex.2014.12.020
